# Signaling and Detoxification Strategies in Plant-Microbes Symbiosis under Heavy Metal Stress: A Mechanistic Understanding

**DOI:** 10.3390/microorganisms11010069

**Published:** 2022-12-26

**Authors:** Yao Liu, Guandi He, Tengbing He, Muhammad Saleem

**Affiliations:** 1 College of Agricultural, Guizhou University, Guiyang 550025, China; 2 Key Laboratory of Plant Resource Conservation and Germplasm Innovation in Mountainous Region, (Ministry of Education) Guizhou University, Guiyang 550025, China; 3 Key Laboratory of Animal Genetics, Breeding and Reproduction in the Plateau Mountainous Region, (Ministry of Education) Guizhou University, Guiyang 550025, China; 4 Institute of New Rural Development, Guizhou University, Guiyang 550025, China; 5 Department of Biological Sciences, Alabama State University Office, 314, 1627 Harris Way, Montgomery, AL 36104, USA

**Keywords:** common symbiotic signaling pathway (CSSP), chelator, transporter, signaling receptor

## Abstract

Plants typically interact with a variety of microorganisms, including bacteria, mycorrhizal fungi, and other organisms, in their above- and below-ground parts. In the biosphere, the interactions of plants with diverse microbes enable them to acquire a wide range of symbiotic advantages, resulting in enhanced plant growth and development and stress tolerance to toxic metals (TMs). Recent studies have shown that certain microorganisms can reduce the accumulation of TMs in plants through various mechanisms and can reduce the bioavailability of TMs in soil. However, relevant progress is lacking in summarization. This review mechanistically summarizes the common mediating pathways, detoxification strategies, and homeostatic mechanisms based on the research progress of the joint prevention and control of TMs by arbuscular mycorrhizal fungi (AMF)-plant and Rhizobium-plant interactions. Given the importance of tripartite mutualism in the plant-microbe system, it is necessary to further explore key signaling molecules to understand the role of plant-microbe mutualism in improving plant tolerance under heavy metal stress in the contaminated soil environments. It is hoped that our findings will be useful in studying plant stress tolerance under a broad range of environmental conditions and will help in developing new technologies for ensuring crop health and performance in future.

## 1. Introduction

With the continuous acceleration of industrialization and urbanization, a series of environmental problems caused by anthropogenic activities such as over-mining of mines, excessive use of pesticides and fertilizers, and massive discharge of man-made waste have negatively affected soil health and crop production [1,2,3,4] Due to their physicochemical properties such as ubiquity, non-biodegradability, toxicity, accumulation, and persistence, the pollution caused by toxic metals (TMs) has attracted extensive attention from scholars at home and abroad [5,6]. Contamination of soil by TMs leads to a decline in soil quality and fertility, loss of microbial biodiversity, destruction of vegetation cover, and reduced crop yield and quality [7,8]. However, unique coping mechanisms have been developed between soil microorganisms and plants to reduce the toxicity of TMs to plants, such as the interactions between microorganisms and plants, which play a vital role in remediation of soils contaminated by TMs [6,9,10,11]. Among the broad network of plant and soil microbes, fungi and bacteria are typical examples of successful co-evolution with hosts since plants adapted to terrestrial ecosystems [12]. The most widespread fungi of all taxa in which extant terrestrial plants and microorganisms coexist are arbuscular mycorrhizal fungi (AMF) [13]. The symbionts formed by AMF and plants have appeared 476 million years ago (such as hornworts, liverworts, lycopods, and ferns), and the fossils of angiosperms prove that the symbionts of Rhizobium and legumes appeared 111 million years ago. It shows that the formation of this symbionts is later than that of AMF symbionts [14]. Therefore, it has been suggested that the symbiotic relationship between legumes and Rhizobium evolved from the symbiotic relationship formed by AMF and plants [15,16].

There is sufficient evidence that AMF and Rhizobium play a significant role in plant coping with TMs stress. For example, all of them have strong uptake capacity for TMs (such as Zn and Cd), which can enhance the TMs content in plant roots and reduce the transfer of TMs to shoots, thereby improving plant growth in TM-contaminated soils [17,18,19]. However, since the discovery of the symbiotic relationship between AMF and Rhizobium and plants, most researches focus on their symbiosis actively participating in plant life activities (such as morphological development and signal transmission and transduction) and analyzed the relationship between symbiosis and life phenomena (such as gene expression regulation and cell signal transduction, etc.) from the molecular level [20,21,22,23,24]. On the module of TM-contaminated soils, according to several studies, the AMF-plant and Rhizobium-plant symbiotic system cannot only alleviate the toxicity of TMs to plants, but also improve the soil contaminated by TMs [25,26,27,28]. With further research, scientists found that AMF and Rhizobium also have a mutually beneficial symbiotic relationship to some extent, for example, the dual effects of AMF and Rhizobium can improve plant nutrient absorption, promote growth, and change soil microbial community dynamics [29,30,31]. These results indicated that the combined effect of AMF and Rhizobium was much more superior than that of a single strain. Although the significance of AMFs and rhizobium interactions on plant growth and stress tolerance is predicted, however, the intensity of the combination effect of different strains could vary [32]. Regrettably, little is known about how the interaction between AMF and Rhizobium affects plant growth and the molecular mechanism of stress, especially when related to TMs, stress has not been reported.

**Figure 1:**
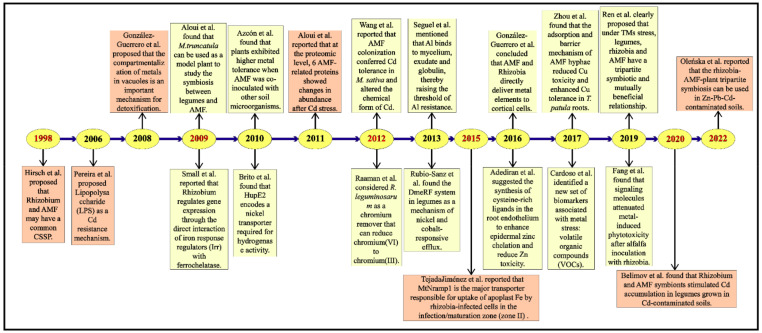
The research history of rhizobia and AMF over the years is summarized. The orange boxes indicate relatively groundbreaking rhizobial or AMF research findings in the field that year. The lemon-yellow boxes represent the current year’s findings of rhizobia and AMF research in this area. Publication time of some key literature on Rhizobium-plant symbiosis and AMF-plant symbiosis and the tripartite symbiosis on TM-contaminated soil of the effects. We have listed representative references of the studies included Hirsch et al. [33], Pereira et al. [34], González-Guerrero et al. [35], Aloui et al. [17], Small et al. [36], Azcón et al. [37], Brito et al. [38], Aloui et al. [39], Wang et al. [40], Raaman et al. [41], Seguel et al. [42], Rubio-Sanz et al. [43], Tejada-Jiménez et al. [44], González-Guerrero et al. [45], Adediran et al. [46], Zhou et al. [47], Cardoso et al. [48], Ren et al. [49], Fang et al. [50], Belimov et al. [51], Oleńska et al. [52].

Ren et al. demonstrated that the interaction between AMF and rhizobia could better prevent and control plant TMs stress [49] Figure 1, and how the symbiosis of AMF and rhizobia can jointly regulate themselves and host plants from being poisoned by TMs and ameliorate soil pollution problems, etc. Clarifying these problems will help us understand the remediation and management of TMs in soil. At a broader level, a better understanding of environmental and genetic cues is essential for the future use of microbial communities to achieve sustainable agricultural growth. Therefore, based on current research advances, this paper focuses on the cooperative symbiosis between AMF, rhizobia, and plants and their interactions, and summarizes the advantages of dual inoculation of AMF and rhizobia on plant growth. The symbiotic interactions between AMF and rhizobia and their mitigation or detoxification strategies for plants in response to TMs were further analyzed. Meanwhile, we also prospose the molecular regulatory mechanisms of plant-AMF-rhizobia interactions to empower plants or microorganisms to reduce the toxicity of TMs. Finally, we propose further research directions for the interactions between AMF, rhizobia, and plants.

## 2. AMF and Rhizobium Co-Mediate Signal Transduction Pathway in Plant Cells

**Figure 2:**
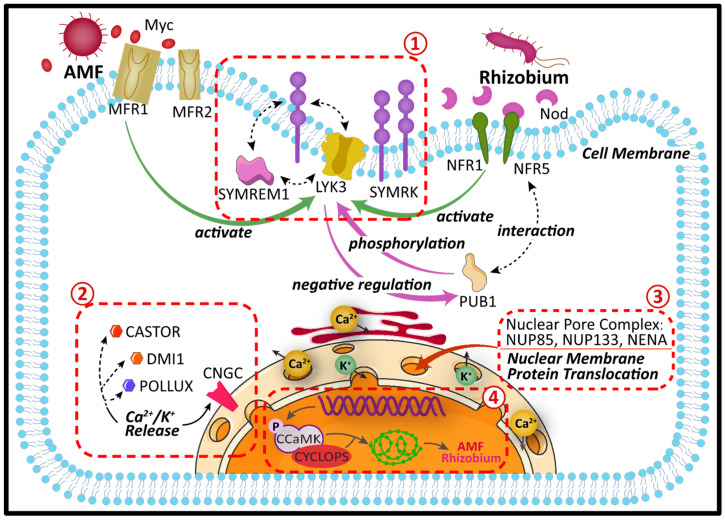
Major components in plant (vacuole omitted) symbiosis with AMF and Rhizobium involved in symbiotic signaling. ①, ②, and ③ represent the three possible signal processes in this pathway (extramembrane signal, extranuclear signal, and intranuclear signal, respectively).

In legumes, some studies have found that mutants with defects in the nodule symbiosis phenotype also have defects in the arbuscular mycorrhizal symbiosis phenotype; this revealed a genetically overlapping association between two different symbiotic systems, arbuscular mycorrhizal symbiosis and nodule symbiosis [15]. The establishment of Rhizobium and AMF symbionts involves several genes/proteins defined by clonally mutated loci and differentially expressed non-functionally characterized genes [53]. Eight genetic loci have been identified to date as components of the Common Symbiotic Signaling Pathway (CSSP) shared by Rhizobium and AMF [33] (Figure 2). The genes/proteins involved in the AMF and Rhizobium symbiotic system are symbiotic receptor kinase (SYMRK) [54], nucleoporins (NUP85, NUP133, NENA) [55,56,57], and ion channel proteins (CASTOR, POLLUX) [58]. These six genes are located upstream of Ca^2+^ spiking signaling. Calcium- and calmodulin-dependent protein kinase (CCaMK) and the DNA-binding transcriptional activator (CYCLOPS) are located downstream of Ca^2+^ spiking signaling [59]. A physical interaction occurs between CCaMK and CYCLOPS, which are responsible for decoding Ca^2+^ spikes and activating several transcription factors located downstream of CCaMK, including nodulation signaling pathway 1 and 2 (NSP1, NSP2) and genes expressed in the early stage of the root nodule [17]. Mutation analysis indicated that LRR protein kinases (SYMRK/NORK) and cation channels (CASTOR/POLLUX/DMI1) are required for Ca^2+^ spiking induction [60]. In addition, when plants feel attacked or injured, chitin signaling molecules secreted by AMF activate the CSSP pathway to participate in the defense after being sensed by LysM receptor-like kinase (RLK) [61]. Downstream signal transduction pathways shape the apoplast intracellular infection pattern that ultimately leads to the coordinated development of a complex bidirectional symbiotic interface found in AMF and Rhizobium.

### 2.1. The Entry Point of the Symbiotic Pathway: SYMRK

Plant cells utilize different molecular mechanisms to facilitate the adaptation of beneficial microorganisms, or to prevent the entry of adverse environments [62]. Symbiosis receptor kinase (SYMRK) is essential for the development of AMF symbiosis and root nodule symbiosis, and it regulates the symbiotic relationship between legumes, Rhizobium, and AMF [63,64,65,66]. The SYMRK is a commensal receptor kinase with leucine-rich repeat (LRR) and extracellular malectin-like domain (MLD). Its subcellular localization is in the cell membrane [67]. It was originally found in *Medicago sativa* and *Lotus. japonicus* and was later found to be conserved in most angiosperms [68,69]. The SYMRK gene is active near the initial junction of the AMF and Rhizobium signaling cascade. Mutations in this gene not only affect nodulation, but also impair mycorrhizalization of clumped mycorrhizae [69]. Remorins are a plant-specific gene family whose member SYMREM1 is involved in legume root nodulation. SYMREM1 interacts with the symbiotic receptor kinases MtNFP, MtLYK3, and MtDMI2 in *Medicago truncatula* and direct homologues of NFR5, NFR1, and SYMRK in *L. japonicus* also interact with SYMREM1 [70]. Indrasumunar et al. used RNA interference (RNAi)-mediated knockdown of GmSymRK gene activity, which showed a significant reduction in nodulation and mycorrhizalization [71]. SYMRK has an MLD, three LRR motifs, a transmembrane structural domain, and a Ser kinase structural domain [61]. Due to its structure and corresponding mutant symbiotic phenotype, SYMRK is speculated to be one of the primary parts of the symbiotic pathway CSSP. Mediating signals generated by the sensing of Myc factor receptors (MFR) and Nod factor receptors (NFR) at the plasma membrane are activated by the CSSP pathway [72]. In this mode, SYMRK has the potential to sense signals from extracellular microbial symbionts either directly or indirectly and transduce that signal through the intracellular kinase structural domain. However, the ligands of the extracellular structural domains have not been identified.

### 2.2. Extranuclear Cation Channels: CASTOR and POLLUX

A family of plant extranuclear cation channels, including DMI1 and its homologs CASTOR and POLLUX [60]. These extranuclear cation channels in legumes generate Ca^2+^ spikes in and around the nucleus, and thence regulate the expression of symbiotic genes and establish a plant-microbe symbiotic relationship [58]. In plant-microbe interactions, Ca^2+^ spikes and oscillations are key to signal specificity: external stimuli cause cytoplasmic spikes when symbiotic microbes trigger nuclear Ca^2+^ oscillations [73]. CASTOR and POLLUX are localized to the nuclear membrane and are prevalent and highly conserved in plants. It has been found that CASTOR and POLLUX play similar roles in symbiotic signaling. However, there seems to be no interaction between these two proteins, and they only form homogeneous ion channels providing an important pathway for ion homeostasis in plant cells. It has been reported that CASTOR and POLLUX are superior to other cations (such as Na^+^ and Ca^2+^) for K^+^ selection [74]. However, Kim et al. uncovered that CASTOR is a better choice for Ca^2+^ than K^+^ or Na^+^, since cytoplasmic/nucleoplasmic Ca^2+^ is required for CASTOR to be activated. This revealed that DMI1, POLLUX, and CASTOR could have functioned as Ca^2+^ release channels in the nucleus, thus negating the previously thought K^+^ channels [58]. The CASTOR, POLLUX, and DMI1 channels contain four transmembrane (TM) fragments followed by a rather large soluble structural domain. Structurally, the cytoplasmic/nucleoplasmic ligand-binding soluble region of CASTOR contains two tandem RCK structural domains [58]. Electrophysiological measurements of recombinant ion channels in lipid membranes and yeast complementation experiments clearly demonstrate that these proteins act as channels for K-permeable cations [72]. The nuclear envelope cyclic nucleotide-gated channel (CNGC) is a Ca^2+^ permeable channel with which DMI1 has been discovered to interact. DMI1, CASTOR, and POLLUX can modulate membrane potential across the nuclear membrane and also regulate nuclear Ca^2+^ release in conjunction with CNGC channels, leading to speculation that they may function as ligand-gated K^+^ channels. Whether the soluble structural domains of these channels contain RCK structural domains to be proven structurally due to the low sequence similarity [74] The soluble domains of CASTOR, POLLUX, and DMI1 modulate channel activity by sensing cytosolic/nuclear Ca^2+^ concentrations, respectively, and thus use Ca^2+^ binding to directly mediate Ca^2+^ release from the nuclear membrane by the RCK gating loop [75].

### 2.3. Co-Utilization of Nucleoporin Channels

When the plant senses diffusible signals from the symbiosis, Ca concentrations in and around the nucleus of root epidermal cells surge causing Ca^2+^ spiking under the action of AMF and Rhizobium [72]. These surges are critical for activating downstream gene expression. Ca^2+^ spiking is an early physiological response to symbiotic signaling. One possible role of nuclear pores in Ca^2+^ spiking is to act as a gate for calcium ions or as a factor for interaction with calcium channels [76]. Nuclear pore proteins are channels that regulate the transport of macromolecules (e.g., mRNA export and protein input across the nuclear membrane) between the nucleus and cytoplasm [77]. Abundant evidence suggests that mutations in the nucleoporin genes NUP85, NUP133, and NENA lead to defects in plant-microbe symbiotic signaling [55,56,57]. For example, in *L. japonicus*, mutant analysis showed that NUP85, NUP133, and NENA may be involved in Rhizobium and AMF symbiosis, and defects in these genes resulted in their inability to induce nuclear Ca^2+^ spiking, leading to a nodulation and mycorrhizal colonization defective phenotype [55]. In addition, these mutants exhibited a stress-sensitive phenotype in Rhizobium symbiosis and AMF symbiosis [78]. Although we do not yet know their specific roles in symbiosis, it is speculated that it may be that NUP is involved in translocation between nuclear membrane proteins [79]. NFR1 and NFR5 are receptor kinases that function upstream of a common pathway in the model legume *L. japonicus*. Presumably, they signal Nod factors into a signal transduction pathway shared with mycorrhizal fungi to achieve symbiotic reciprocity [76]. It has also been reported that MCA8 is a calcium ATPase, which is an essential component of Ca^2+^ spiking. MCA8 may be involved in reloading Ca^2+^ into the nuclear membrane and ER lumen, thereby replenishing Ca^2+^ stores, and readjusting Ca^2+^ concentrations in the cytoplasm and nucleoplasm. In general, when cells are stimulated by the outside world, the concentration of intracellular Ca^2+^ increases, and Ca^2+^ causes a calcium signal in the nucleus to generate a response through the nuclear pore complex pathway [75]. Whether other cations can also be regulated under the action of these nuclear pore proteins deserves further verification and analysis by researchers.

### 2.4. Intranuclear Signaling: CCaMK and the Coiled-Coil Protein CYCLOPS

The CCaMK is currently considered a mechanism for encoding and decoding calcium signals, and its regulation is complex, including positive and negative regulation facilitated by autophosphorylation at two conserved sites [78]. Studies have shown that the primary function of signaling components upstream of calcium spikes is to activate CCaMK [80]. Whether in yeast or plants, in vitro assays indicate that CCaMK and CYCLOPS can interact [72], and CCaMK can both phosphorylate the substrate and CYCLOPS to form an ancient signal transduction complex [81]. This complex is specifically targeted for fungal and Rhizobium infection, and the formation of root nodule organs may require the involvement of other CCaMKs and substrates that have not yet been identified [82]. Transients in calcium concentration have been reported to correspond to a specific symbiotic system where CCaMK interacts with the DNA-binding transcriptional activator CYCLOPS [83]. Several transcription factors (NSP1, NSP2, RAM1, and RAD1) are activated downstream of CCaMK and CYCLOPS and determine whether plants participate in AMF and Rhizobium symbiosis [63]. In conclusion, the symbiosis between Rhizobium and AMF involves complex network signaling, and it’s signaling pathway needs further verification and research.

## 3. Detoxification Regulation Strategies of Rhizobium Symbiosis and AMF Symbiosis under Toxic Metal Stress

We know that plants will form beneficial relationships with AMF or Rhizobium to resist adverse environments based on the above discussion. Rhizobium and AMF have evolved various mechanisms to protect themselves and plants from toxic metal poisoning (Table 1). Plants adapt avoidance strategies as the first step in dealing with metal poisoning. Metals are prevented from entering plant roots by limiting the uptake of the metal by the soil or by removing it [84]. If the avoidance strategy fails and TMs enter the interior of plant tissues, tolerance mechanisms of detoxification are activated, including metal ion transport, organic acids, polysaccharides, and metallothioneins (MTs) to achieve intracellular complexation or chelation of metal ions [85]. Ultimately, if all measures fail to defend against the damage and the plant is overwhelmed with the toxicity of the TMs, antioxidant defense mechanisms will be activated [86]. Here, we attempt to introduce a brief overview of the mechanisms of fungal/bacterial cell wall adsorption and siderophore chelation, polysaccharide species (exopolysaccharide EPS), MTs, and redox, respectively. It is worth considering whether the interaction between AMF and Rhizobium involves the above mechanism or other mechanisms to enhance the plant’s response to TMs damage. Alterations in the content of TMs in plants and increased plant tolerance may also be associated with extensive changes in AMF and Rhizobium gene expression and protein synthesis induced by the symbiosis itself. In addition to affecting TMs accumulation and plant gene expression, the establishment of AMF and Rhizobium symbiosis may also alter the allocation of TMs within plants.

### 3.1. Cell Wall Adsorption and Siderophore Chelation

TMs enter root cells through plasma membrane transporters or channels (including ZIP, NRAMP transporters, and Ca^2+^ channels), and the cell wall is the first barrier for TMs to enter plant root cells [73,116,117]. Metal ions can be absorbed by the cell wall components of Rhizobium and AMF, peptidoglycan and chitin, or extracellular mucus. The AMF then transports the adsorbed metal ions into its own vacuoles for compartmentalized isolation. [34,118,119]. Rhizobium immobilizes metal ions on the cell surface through mechanisms such as complexation, coordination, and ion exchange, thereby preventing plant root cells from absorbing excess metals from the soil and causing toxicity [120,121]. For example, phosphate and carboxyl groups associated with Rhizobium cell envelope proteins and phosphorylated biopolymers (such as teichoic acid, surface proteins, and peptidoglycan) mediate uranium (Ur) biosorption, leading to greater accumulation of Ur in the cell envelope [122]. The mycelium on the surface of the AMF structure adsorbs metal ions and deposits them on the cell wall, and then reduces TMs from one form to another by positively charged particles, including amino acids, thiol groups, and glutathione, etc., to reduce toxicity to plants [123,124]. This illustrates the importance of microbial cell walls/vacuoles as key mediators for metal adsorption by microorganisms.

Siderophores are substances that chelate iron secreted by microorganisms at low iron concentrations and are mainly found in microorganisms such as bacteria and fungi. It can be divided into three categories: catecholates (existing only in bacteria), hydroxamates (produced by bacteria and fungi), and carboxylates (rhizobactin produced by Rhizobium meliloti), Fungi tend to synthesize hydroxamate-type siderophores [125,126]. The most commonly reported role of siderophore is to chelate iron, and its process includes the main co-transport superfamily transporter MFS and the participation of drug resistance, nodulation, and cell division superfamily RND [127]. Under conditions of limited iron content, siderophores act as iron solubilizers. It usually forms a 1:1 complex with Fe^3+^, which is then taken up by the fungal/bacterial cell membrane, where Fe^3+^ is reduced to Fe^2+^ and released from siderophores into the cell [127]. In addition to iron, siderophores can also complex with other environment-related metals such as Al, Cd, Cu, Pb, and Zn, as well as radionuclides [128,129,130,131]. It was found that the ability of the siderophore to complex these metals mainly depends on the stability constant of the complex formed by the siderophore and the metal [132].

**Figure 3:**
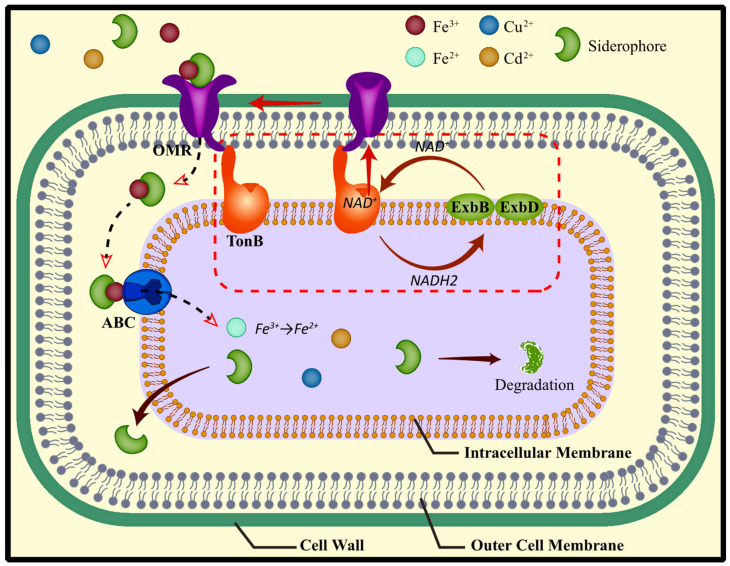
Mechanism of siderophore chelation of metals. Each metal-siderophore complex is recognized by a specific outer membrane receptor (OMR), and the complex is transported into the intracellular membrane by the transporter ABC, where the metal ion is converted to a less toxic ionic form. When the siderophore mission is completed, it will be degraded or transported to the cell wall for standby. The OMR interacts with the inner membrane proteins TonB, ExbB, and ExbD to facilitate the uptake of metal-siderophore complexes.

Each iron-siderophore complex is recognized by a specific outer membrane receptor (OMR), and although OMRs are extremely diverse, different bacterial species and different siderophore classes have different receptors [133]. OMR interacts with the inner membrane protein TonB to facilitate uptake of the iron-siderophore complex. The current model suggests that TonB transfers the energy generated by the inner membrane proteins ExbB and ExbD and the electrochemical proton motive force generated in the periplasm during normal cellular respiration to the OMR, resulting in a conformational change in the OMR. The conformational change of the OMR facilitates the intracellular transport of iron-siderophore complexes (Figure 3) [133,134,135,136]. Studies have shown that inoculating plants with siderophore-producing bacteria and fungi can reduce the uptake of TMs. For example, Ren et al. found that bacteria (such as *Pseudomonas fluorescens*, *Rhizobium,* and *Azospirillum lipopherum*) can enhance the tolerance of *M. sativa* to high concentrations of Cu [137]. The results of Sepehri and Khatabi showed that Rhizobium (*S. meliloti*, possessing siderophore capacity) alone or in combination with fungi (*P. indica*) to improve the potential of Fe transport from underground to shoots may also help better coping with Cd Stress in Plants [138]. However, until now, there are few reports that specifically describe whether and how siderophores are released by AMF. The cause may be that the technology for extracting and isolating large amounts of siderophores cannot be achieved under sterile conditions, but this cannot rule out the possibility that AMF does not produce siderophores. Glomuferrin is an iron chelating compound (carboxylate siderophore) present in the roots of *Tagetes patula* colonized by *Glomus spp*, discovered by Winkelmann [139]. This evidence well demonstrates the importance of siderophores and plant or microbial cell walls in response to TMs toxicity.

### 3.2. Gating Guards: The Extracellular Polymer EPS

In recent years, scientists believe that Extracellular polymeric substances (EPS) are biopolymers (such as bio-films, bio-flocs, and bio-particles) produced during the symbiosis and self-growth of various microorganisms (microalgae, bacteria, fungi) and plants caused by the environment, which are located on or outside the cell wall of microorganisms. EPS can be loosely attached to the cell surface, or microbes can be embedded in EPS [140,141]. Its production is one of the most common strategies used by microorganisms to protect themselves and plant cells from adverse environmental conditions (Table 2). *Burkholderia cepacia* (belonging to the genus Pseudomonas) isolated from TMs-contaminated soil can produce EPS and bind nonspecifically to cations. This results in the adsorption of extracellular Cd by EPS, and thus the ability of EPS to chelate metal ions reduces the bioavailability of Cd [142,143]. In addition to this, it has been reported that under the stimulation of Zn^2+^ ions, Rhizobium produces EPS and other biofilms, which enhances the cellular hydrophobicity [144]. Wu et al. believed that AMF produced a large number of EPS-like substances on its surface, which could adsorb and reduce Cr (VI) or Cr (III)-phosphate analogs [145]. These reports suggest that TMs can not only induce EPS synthesis in bacteria and fungi in response to TMs stress, but also the generated EPS can significantly adsorb these metals, thereby reducing the toxicity of TMs to plants and microorganisms themselves. Fungal external mycorrhizal hyphae can interact directly or indirectly with bacteria in soil by altering the physiology and root exudation patterns of the plant host [146]. It has been reported that the mycelium of AMF may be the preferred microhabitat for microorganisms. Many bacteria, including Rhizobium, can colonize and form biofilms on AMF hyphal surfaces to varying degrees in response to environmental stress induced by TMs and trap metal ions in their EPS [147]. At present, there are few studies on the interaction between AMF and Rhizobium on EPS adsorption or binding of toxic metals, which may be due to the difficulty in extracting and isolating EPS-producing functional strains from AMF. This limitation brings a difficult task for plants to combine EPS-producing AMF and Rhizobium to remediate soil contaminated with toxic metals.

**Figure 4:**
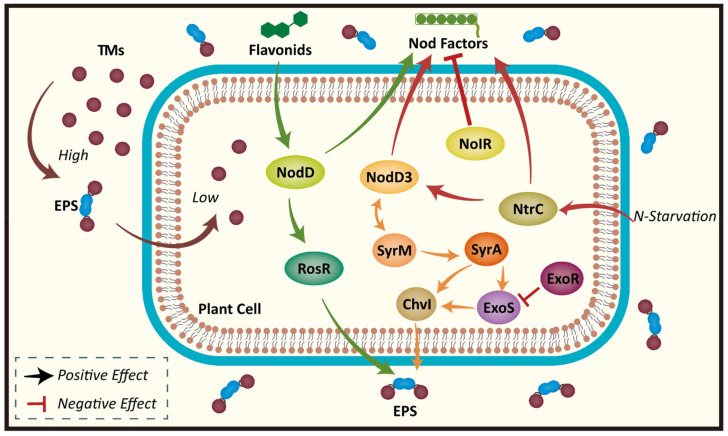
Schematic diagram of EPS binding to TMs in soil under the regulation of relevant genes. Different colored arrows play an active role in the process, and T-shaped arrows indicate an inhibitory role in the process.

Microbial production of EPS is a protective response to stress for survival and growth in a metal contaminated environment. According to reports, molecular regulatory pathways currently exist for EPS adsorption and binding of TMs in rhizobia and AMF (Figure 4). Specifically, plant roots secrete flavonoids, which at the right time trigger the expression of nodulation genes by activating the rhizobiuml LysR regulator NodD, resulting in the production and secretion of Nod factors. Simultaneously, flavonoids and NodD activate RosR to enhance EPS syntheses [156]. Additional RosR promoted an increase in the number of EPS synthesis and enhanced nodulation and symbiosis [157,158]. ExoR/ExoS/ChvI form a major regulatory circuit that regulates exo gene expression, with ExoS activating ChvI through phosphorylation, which in turn activates exo gene expression [159,160]. ExoR inhibits the synthesis of ExoS thereby suppressing the production of EPS [161,162]. In addition, when plants are nitrogen deficient, the nitrogen metabolism regulatory protein NtrC is induced and the expression of NodD3 is activated by SyrM and vice versa, enhancing the synthesis of Nod factors. In addition to NodD3, SyrM also activates the expression of syrA, which in turn stimulates the production of EPS [156,163]. The large amounts of EPS produced by Rhizobium under unfavorable environments can sequester TMs in soil and achieve the reduction of TMs toxicity to host plants.

### 3.3. Binding of TMs to Metallothioneins

**Figure 5:**
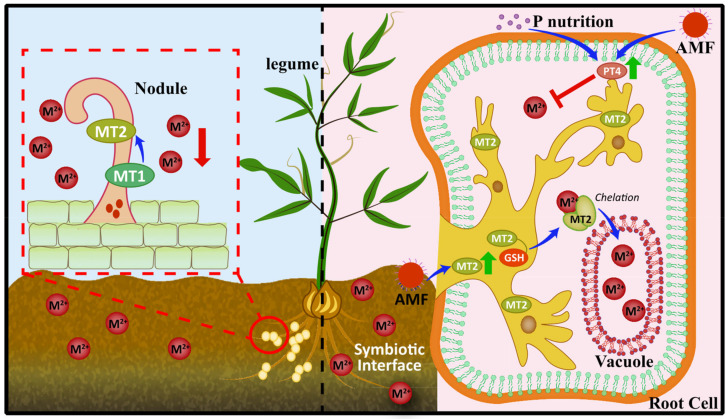
Brief overview of the mechanisms by which MTs enhance plant tolerance to TMs. MT1 and MT2 enhanced the tolerance of root nodules to TMs. At high content of As, AMF enhanced plant phosphorus (P) nutrition, promoted the expression of phosphorus transporter PT4, thereby inhibiting toxic metal ions, and stimulated the expression of MT2 and GSH in plant roots, sequestering more As in roots, and reducing As translocated into root cell vacuoles for storage and detoxification. The red spheres indicate metal ions. MT1 and MT2 denote metallothioneins.

Metallothioneins (MTs) are thermostable metal-chelating proteins produced by microorganisms and plants in metal-rich environments, capable of binding with high affinities such as zinc, copper (Cu), and lead (Pb), etc. [115,164]. Once TMs enter the symbionts, they induce plants to produce MTs. MTs in plant cells combine with TMs to form TMs crystals or precipitates, reducing the activity of metal ions, and thereby reducing or relieving the toxicity of TMs (Figure 5) [165]. Current evidence suggests that MTs enhance the tolerance of root nodules to TMs during legume Rhizobium symbiosis. The results of Tsyganov et al. showed that in the symbiosis of *Pisum sativum* and Rhizobium, MT1 and MT2 were Cd-tolerant to nodules formed by Rhizobium [166]. Pérez-Palacios et al. heterologously expressed Atmt4a into *M. truncatula* and induced greater tolerance to Cu [167]. To our knowledge, there are two MTs proteins, MT1 and MT2, that specifically bind TMs ions in plant and AMF symbiosis. For example, yeast functional complementation analysis clearly demonstrated that *Gigaspora margarita* (a type of AMF) MT1 can chelate metal ions, thereby resisting the toxicity of Cd, Cu, and Ni to yeast cells [168]. For arsenic (As), there is good evidence that AMF enhances plant phosphorus (P) nutrition and promotes plant growth, thereby diluting As uptake by plants [169]. In soils with high As content, *Rhizophagus irregularis* stimulated the expression of *MsMT2* and *MsGSH* in *M. sativa* roots, sequestered more As in roots, and translocated As into root cell vacuoles for storage and detoxification [170]. Despite the diverse functions of MTs in microbes and plants, little is known about how MTs function in the Rhizobium/AMF-plant symbiotic association. This work needs to be further studied by relevant experts.

### 3.4. Metal Detoxification by Redox

The primary toxicity of TMs is due to their high-affinity for sulfhydryl groups (SH), which inactivate major metabolic enzymes and consequently interfere with cellular metabolism, and adversely affect the integrity of the cellular plasma membrane, thereby inhibiting plant life activities [171,172]. However, the binding of Cd to SH renders the Cd ions harmless to cells by chelating with metal detoxification ligands, thereby converting them to a more harmless form [173]. Currently, glutathione (GSH: L-γ-glutamyl-L-cysteinylglycine) and phytochelates (PCs) are one of the general mechanisms for complexing and detoxifying metal ions [174]. GSH is a substrate for sulfur-containing tripeptide thiols and cysteine-rich PCs (γ-Glu-Cys)2-11-Gly. Glutathione S-transferases (GSTs), with the help of GSH, can reduce peroxides and produce scavengers of cytotoxic and genotoxic compounds, thus achieving degradation of cytotoxicity to protect plants [175].

**Figure 6:**
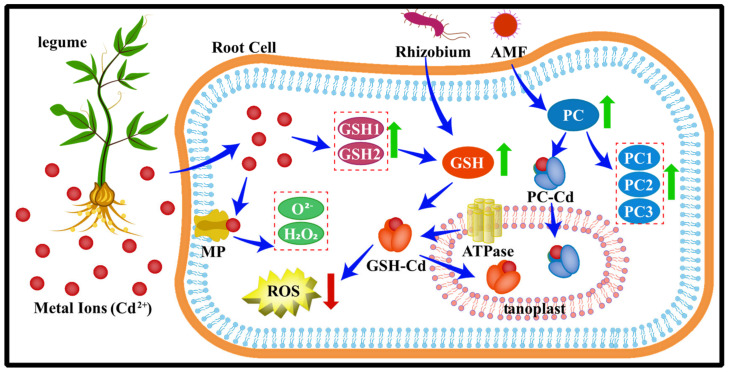
After the entry of metal ions (mainly representing Cd^2+^) into cells, GSH1 and GSH2 genes (two enzymes involved in encoding GSH synthesis: γEC synthase and GSH synthase) were up-regulated under the action of Cd. GST catalyzes the nucleophilic attack of GSH on the electrophilic group of Cd to form a GSH-Cd complex. PC is synthesized from GSH in a reaction catalyzed by PC synthase. The tonoplast ATPase sequesters GSH-Cd and PC-Cd complexes in the vacuole, thereby helping to mitigate Cd toxicity and avoid cellular metabolic toxicity. In addition, proteins on cell membranes interact with metal ions to reduce oxidative stress damage in plants. The green arrow shows an increase in the content and the red arrow shows a decrease in the content in the figure. The blue arrow shows the protective activities that occur within a cell to reduce the toxicity of a metal ion when it enters.

It has been reported that the formation of Cd-GSH complexes is a mechanism of Cd tolerance in Gram-negative bacteria Rhizobium legumes [176] (Figure 6). The electrophilic group of Cd can be attacked by tripeptide glutathione (GSH) under the catalysis of glutathione transferases (GSTs), and are mainly involved in the formation of the GSH-Cd complex, thereby the Cd tolerance in the symbiotic relationship between pea and Rhizobium was determined [173]. Phytochelatins (PCs) are enzymatically synthesized from GSH by PC synthase in the presence of metal ions [177]. It has been shown that AMF inoculation can increase the content of PCs to form Cd-PCs complexes through chelation. PCs react with Cd via glutathione S-transferase in the cytoplasm, which then chelates them into the vesicles. The subsequent sequestration of PC-Cd in vacuoles by tonoplast ATPases helps to alleviate Cd toxicity and avoid cellular metabolic toxicity, promote plant growth, and reduce cadmium accumulation in maize [178]. In the study by Most and Papenbrock, it was proposed that PC synthesis is stimulated by Cd stress and that PC rapidly forms “low molecular weight” and “medium molecular weight” complexes with Cd to form PC-Cd complexes, which are highly polymeric and acquire acid-unstable sulfur (S^2−^) in vesicles and form a high affinity for Cd ions “high molecular weight” complexes with high affinity for cadmium ions. Thus, the “high molecular weight” complexes with highly stable S^2−^ groups seem to play a decisive role in Cd detoxification [179]. Furthermore, at the cellular level, metals can interact with membrane proteins, leading to lipid peroxidation and oxidative stress by disturbing the redox balance in plants [48]. For example, it was found that M. sativa root inoculation with Rhizobium can control the levels of O^2−•^ and H_2_O_2_ in plants and regulate the expression abundance of SOD-related genes, thereby reducing the oxidative stress of excess Cd and Cu [48,112].

## 4. Plant-AMF-Rhizobium Work Together to Regulate Metal Homeostasis

**Figure 7:**
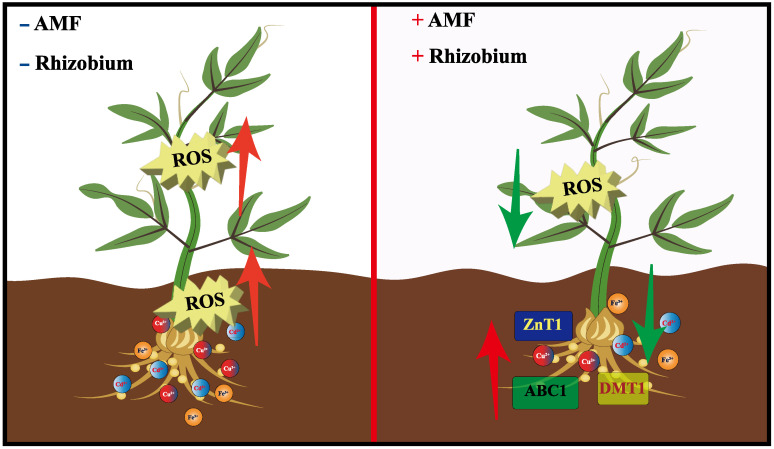
Effects of double inoculation of Rhizobium with AMF on plant growth. After double inoculation with AMF and Rhizobium, the content of ROS in plants decreased, the expression of ABC1, DMT1, and ZnT1 genes increased, which promoted the transport and absorption of Cd^2+^, Cu^2+^, and Fe^3+^, thus achieving the effect of reducing the concentration of TMs.

The tripartite symbiotic relationship between Rhizobium, AMF, and plants further demonstrates the complexity of microbial interactions that enhance plant resistance to environmental stress [49,180]. Figure 7 shows the changes in the reactive oxygen species (ROS) in legumes after double inoculation with AMF and Rhizobium. However, due to the obligate symbiosis of AMF and its multinucleate nature, little progress has been made in the research on the relationship between plant-AMF-Rhizobium, but it has always attracted people’s attention. Studies have shown that some legumes in inactivated soil can only develop nodules in the presence of AMF [181]. Later, it was found that the presence of AMF can significantly increase the nodulation number of Rhizobium, and it is believed that this may be caused by the fact that AMF provides sufficient P for the nodulation process of Rhizobium [182]. The current study shows that Rhizobium and AMF interact and help to improve the tolerance or repair efficiency of plants to TMs. For example, the best uranium (Ur) removal rate was observed with double inoculation of AMF and Rhizobium in soil, reaching 50.5–73.2%. After double inoculation, the expression of phytochelatin synthase (PCS) gene was increased, and the content of organic acid was also significantly increased [49]. Belimov et al. found that inoculation with AMF and Rhizobium and PGPR reduced Cd concentrations in shoots of two pea genotypes (wild-type SGE and mutant SGECdt) [51]. So far, tripartite symbiosis has attracted attention in the remediation of TMs-contaminated soil. However, some studies have also shown that TMs in soil interfere with the establishment of the symbiotic relationship between AMF and Rhizobium [183]. Table 3 summarizes the effects of co-inoculation on plant tolerance to TMs in recent years. However, there are still few reports on the specific mechanism involved in tripartite symbiosis. Mycorrhizal exudates have obvious chemotactic effects on root bacteria and pathogenic bacteria of most host plants [184,185]. However, the research on whether AMF contributes to the chemotaxis of Rhizobium and is beneficial to the infection of Rhizobium and the improvement of nitrogen fixation is still not thorough enough, especially the research on reducing the toxicity of TMs is even less. We currently have a limited understanding of the effects of Rhizobium and AMF co-inoculation on metal degradation in TMs-contaminated soils.

### 4.1. Effects of the Synergistic Antagonistic Relationship between Rhizobium and AMF on Plants

**Figure 8:**
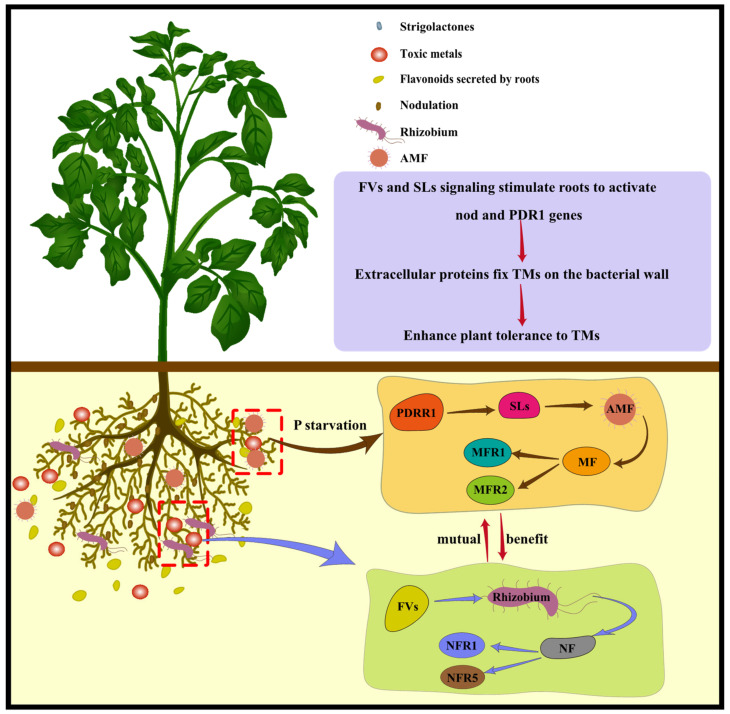
Simplified diagram of flavonoids and solanum lactones bound to TMs secreted by host roots. MF: Myc factors (MFR1 and MFR2). NF: Nod factors (NFR1 and NFR5).

Most plants constitutively release diffusible signaling molecules, strigolactones (SLs), from roots that stimulate AMF [190]. SLs stimulated the roots of *Petunia hybrida* to induce abundant PDR1 expression under the action of the ATP-binding cassette subtype G (ABCG) transporter [191]. In contrast, the PDR1 is preferentially expressed during P starvation, a condition that favors AMF growth, activates AMF to secrete Myc factor, and is received by the receptors for Myc factor in plant cells, MFR1 and MFR2, for this signal [192]. Flavonoids (FVs), regarded by Rhizobium as diffusible inducers, produce a specific symbiotic signal (this is Nod factors, NFs) [193]. These signals are received by dedicated NF receptors NFR1 and NFR5 localized to the plasma membrane [194]. It was found that root secretions produced relatively more accumulated Nod factors during the early stages of plant growth (early AMF infestation) and relatively less of such signaling substances during later AMF infestation [195]. AMF may secrete a certain amount of Myc factor at the early stage of infestation to inhibit the accumulation of Rhizobium to the host plant, while the signaling substances produced at the middle stage of infestation changed and the antagonistic effect gradually decreased, showing a progressive synergistic effect on rhizobium [196]. Although inoculation with AMF showed negative tropism to Rhizobium at the early stage of symbiosis formation, and the two had some antagonistic effects [197]. However, it showed positive tropism to Rhizobium at the later stage of infestation, and the mycorrhizal secretion secreted by the symbiosis could induce more Nod factor production by Rhizobium, which could largely increase the number of nodules of Rhizobium, and then improve the nitrogen fixation capacity and metal toxicity resistance of Rhizobium. At the same time, rhizobium contributes to AMF symbiosis formation when they are induced to produce more Nod factors and the concentration of effective Nod factors increases, which can accelerate the infestation efficiency of AMF [198]. In this way, the signaling substances between AMF and Rhizobium interact and complement each other, making them synergistic and mutually beneficial in the plant-AMF-Rhizobium symbiosis (Figure 8).

### 4.2. AMF-Rhizobium Activates Plant Transporter Proteins to Enhance Metal Tolerance

AMF and Rhizobium can get along well because both microsymbionts induce a common signaling cascade to bind to the host plant [198]. Expression of *M. truncatula* ABCG3 and RAM2/GPAT was strongly induced after AMF inoculation, and their induction required the GRAS structural domain transcription factor RAM1 [199]. PvUPS1 and GmUPS1 transporter proteins are members of the urea permease (UPS) family, and their translocation from the nodal cortex and vascular endothelium to the nodal xylem lumen contributes to the export of urea from the nodes to provide nutrients for plant requirements [200].

The formation of symbiosis leads to the internalization of mycorrhizal fungi and inter-rhizosphere bacteria in the root cortex cells of plants where they are surrounded by plant-derived membranes called symbiosome membranes (SM) [201]. Symbiotic sulfate transporter protein 1(LjSST1) localizes to the SM and transports sulfur into the symbiosis space (SS) [202]. Copper (Cu) transporter protein (COPT1) is a Rhizobiuml nodule-specific metal transporter protein, which is expressed throughout the growth phase of *M. truncatula*. Mutations in MtCOPT1 may have indirect effects on nitrogen fixation and plant physiology under symbiotic conditions between Rhizobium and AMF, which may be caused by a Cu-dependent functional defect in Rhizobium [203]. Iron (Fe) is a key cofactor for nitrogen fixing enzymes, hemoglobin, and other proteins essential for cellular homeostasis [204]. One of the strategies for plants to acquire iron from the environment is to initially reduce Fe (III) to Fe (II) by Fe (III)-chelatase reductase on SM, and then reabsorb Fe (II) [205]. Members of multiple protein families may be responsible for trans-SM transport of Fe (II). For example, GmDMT1 is localized on the SM in nodules and is the only Fe (II) transporter protein currently characterized in root nodules that are involved in Fe (II) transport and iron homeostasis in root nodules to support symbiotic N_2_ fixation [206]. In addition, a major role of MATE67 in the citrate efflux from nodule cells in response to iron signaling has been reported. This efflux is necessary to ensure the solubility and mobility of Fe (III) in the plasmatic ectodomain and its uptake by nodule cells [207]. The transporter protein GintZnT1, isolated from *Glomus intraradices*, transports Zn (II) out of the cytoplasm and plays a role in the compartmentalization of the Zn region and protection of G. intraradices from Zn stress [208]. The results of González-Guerrero’s study suggest that the accumulation of TMs in AMF vesicles implies the presence of many metal transport proteins involved in loading this organelle [35]. The expression of GintABC1 was up-regulated with increasing Cu and Cd levels and is the first ABC transporter protein reported to be involved in Cd and Cu detoxification in AMF so far [209]. In addition to the above-mentioned transporter proteins, the AMF genome encodes members of the natural resistance-associated macrophage protein (NRAMP), yellow stripe-like (YSL), and vesicular iron transporter protein (VIT) transporter families that show high expression in rhizobiuml nodules. They may play a role in the transport of Fe and other metal ions across the SM.

## 5. Conclusions and Prospects

The great potential of Rhizobium and AMF in mitigating stress caused by TMs in plants is evident. In turn, plants establish beneficial associations with Rhizobium and AMF to facilitate access to nutrients that limit plant growth. In the symbiotic interaction, because the two organisms cooperate at such a close distance, they only use one layer of membrane, a thin cell wall, and some interstitial material to separate their cytoplasm. Consequently, the same symbiotic pathway must exist. From the initial recognition of intracellular regulation to the establishment of the symbiotic interface, symbionts participate in the exchange of nutrients at the symbiotic interface. Therefore, this paper reviews the detoxification mechanism of TMs by Rhizobium and AMF in the body or the host, and the roles of related proteins in the possible common signaling pathways of the two. The current study of mutualistic root symbiosis in legumes initially revealed how secreted signaling molecules by microorganisms activate the symbiotic signaling pathway of CSSP, leading to significant cellular remodeling required to control bacterial or fungal entry into root cells and tissues. Namely, Rhizobium-like LCOs may be universal microbial signals recognized by plants bearing other microsymbionts such as AMF and filamentous Frankia. However, recent studies have shown that this is clearly not the whole story, as non-lipid short-chain COs can also signal AMF. Therefore, major future challenges include identifying novel plant receptors for Rhizobium and AMF signaling, and gaining a deeper understanding of how host receptors can differentiate symbiotic and pathogenic elicitors. On the other hand, how the common pathway CSSP activates Rhizobium and AMF to cope with the stress of TMs on plants is also worthy of future research. Beyond that, to understand the ecological significance and evolutionary consequences of symbiotic relationships, whether it is necessary to incorporate the effects of multiple symbionts on a single host is a question worthy of further study.

## Figures and Tables

**Table 1:** Investigation of various TMs in AMF and Rhizobium-assisted phytoremediation over the years.

Types	Host Latin Name	Repaired Heavy Metals	The Mechanism of Repairing HMs	Citation
Types of Mycorrhizal				
*Rhizophagus irregularis*	*Malus pumila*	Cd	Changes in AMF colonization rate led to up-regulation of MdGH3-2 and MdGH3-12 genes.	[87]
*Claroideoglomus etunicatum*	*Zea mays and Trifolium repens*	La, Cd	AMF mycelium binds to HMs, chelates substances to fix HMs, mediates antioxidant activity, and regulates the distribution of HMs in host plants.	[88]
*Claroideoglomus etunicatum*	*Zea mays* L.	La	AMF changes the structure of the rhizosphere microbiome through root signals to enhance the tolerance of corn to La.	[26]
*Rhizophagus irregularis*	*Brachiaria eruciformis* (J. E. Smith) Griseb.	Cr	AMF protects plants from oxidative stress induced by hexavalent chromium by changing the metabolic processes of plants.	[89]
*Funneliformis mosseae*	*Zea mays*	Pb, Zn, Cd	Phyto-stabilization	[90]
*Funneliformis mosseae*	*Sphagneticola calendulacea*	Cd	AMF symbiosis enhances the transport of Cd to the cell wall and the conversion of Cd to a less toxic chemical form, reducing the phytotoxicity of Cd.	[91]
*Glomus mosseae*	*Pisum sativum*	Pb, Cd	AMF increases plant’s enzymatic and non-enzymatic defense strategies.	[92]
*Rhizophagus irregularis*	*Medicago sativa*	Pb	Ri transfer Pb to the planted root segment and be isolated and isolated in the root.	[93]
*Glomus mosseae*	*Lycopersicon esculentum*	Cd, Pb	AMF symbiosis for better growth, chlorophyll synthesis, and stronger osmotic regulation and antioxidant defense mechanisms.	[94]
*Rhizophagus irregularis*	*Medicago sativa*	Pb	AMF inoculation reduces the content of water-soluble Pb complexes.	[95]
*Rhizophagus fasciculatus;Glomus agregatum*	*Zea mays*	Cd, Cr, Ni, Pb	Plant extracts.	[96]
*Diversispora spurcum*	*Cynodon dactylon*	Pb, Zn, Cd	AMF increases nutrient absorption to reduce the translocation of HMs to buds to improve the growth of bermudagrass.	[97]
*Rhizophagus irregularis*	*Glycine max*	Cd	AMF symbiosis reduces the toxicity of Cd in soybeans by enhancing P nutrition and up-regulating the expression of AMF-inducible GmPTs and GmHMA19.	[98]
*Funneliformis mosseae*	*Zea mays*	Cd	AMF increases the proportion of large aggregates and reduces the leaching of Cd in the soil.	[99]
*Glomus clarum;* *Glomus monosporum; Gigaspora nigra*	*Trigonella foenum-graecum*	Cd	Phyto-stabilization.	[100]
*Rhizophagus irregularis; Claroideoglomus claroideum*	*Medicago sativa*	Cd	Reduce the absorption of cadmium by *Medicago sativa* growth in cadmium contaminated soil.	[101]
*Rhizophagus irregularis*	*Phragmites australis*	Cu	AMF can promote the growth and photosynthesis of *P. australis* under copper stress.	[102]
*Rhizophagus irregularis*	*Phragmites australis*	Zn, Cd	AMF uses the activity of antioxidant enzymes to enhance the tolerance of *P. australis* and the ability to enrich Zn and Cd	[103]
*Rhizophagus intraradices; Funneliformis mosseae*	*Robinia pseudoacacia*	Pb	AMF reduces the lead concentration in host leaves, accumulates biomass, and increases photosynthetic pigment content.	[104]
*Rhizophagus intraradices*	*Triticum aestivum*	As	AMF symbiosis increased the concentration of photosynthetic pigments, enhances Hill reaction activity, regulates the activity of various enzymes in leaves, and restores As-mediated changes in sugar metabolism.	[105]
Types of Rhizobium				
*Medicago lupulina*	*Sinorhizobium meliloti*	Cu	The total amount of Cu uptake by shoots and roots of inoculated plants was significantly increased, and the increase in roots was much higher than that in shoots, which reduced the translocation factor and contributed to the stability of Cu plants.	[106]
*Rhizobium pusense*	*Macrotyloma uniflorum*	Cr	Plants inoculated with *R. pusense* showed tolerance to Cr (VI), a large accumulation of Cr (VI) in roots, and a decreasing trend of reactive oxygen species and antioxidant enzymes.	[107]
*Sinorhizobium meliloti*	*Medicago sativa*	Cu	Rhizobium inoculation enhanced copper tolerance by affecting copper uptake, modulating antioxidant enzyme activity and ascorbic acid-glutathione cycle, and affecting the expression of PC biosynthesis-related genes in rice.	[19]
*Mesorhizobium loti*	*Robinia pseudoacacia*	Pb/Zn/Cd/Cu	Inoculation with *M. loti* significantly increased shoot biomass, and TMs were mainly accumulated in roots through plant extraction and plant stabilization.	[108]
*Rhizobium pusense*	*Brassica oleracea*	Pb/Cd	The concentrations of Pb and Cd in *B. oleracea* decreased significantly after inoculation, and the biomass of the plants also increased significantly.	[109]
*Rhizobium sp*	*Lens culinaris*	Pb	Inoculated plant roots accumulated more Pb than shoots and decreased Pb uptake by plants, suggesting that this symbiotic relationship should be investigated for plant stabilization of lead-contaminated soils.	[110]
*Rhizobium sullae*	*Sulla coronaria*	Cd	Symbiosis of host plants with Rhizobium increases plant biomass and increases Cd uptake, especially in roots.	[111]
*Rhizobium leguminosarum*	*Vicia faba*	Cu	*V. faba* inoculation with Rhizobium can help relieve copper stress and plant stability under hydroponic conditions.	[112]
*Sinorhizobium*	*Lens culinaris*	Cd	*L. culinaris* plant-Rhizobium interactions were the most tolerant to Cd.	[113]
*Rhizobium leguminosarum*	*Trifolium repens*	Zn/Pb/Cd	TMs have specific selective properties for genotypes of Rhizobium, conferring tolerance to TMs in plants.	[114]
*Rhizobium metallidurans*	*Anthyllis vulneraria*	Zn/Pb	The thickening of the cell wall and the synthesis of phenolic substances in the vacuole resist the oppression of the plant by the metal.	[115]

**Table 2:** Metal-binding potential of EPS produced by Rhizobium and AMF.

EPS Producer	Adsorbed Metal	Remark	References
Rhizobium and AMF			
*Bradyrhizobium japonicum* USDA110	Mg (II), Fe (III)	Mg (II) and Fe (III) bind to positively charged EPS or react with hydroxyl groups.	[148]
*Bradyrhizobium japonicum* E109	As (III)	Under As (III) treatment, EPS content was significantly increased, which induced biofilm formation.	[149]
*Sinorhizobium meliloti*	Hg (II) and As (III)	EPS type II *exoY* strains pump As (III) from plant cells to the extracellular, thereby increasing As (III) metal resistance.	[150]
*Rhizobium leguminosarum bv. trifolii*	Zn (II)	Zn (II) stimulates the production of EPS and biofilms, and EPS promotes the sequestration of extracellular metals, limiting the influx of metals.	[144]
*Pseudomonas aeruginosa*	Pb (II)	EPS utilizes an ion exchange mechanism to displace intracellular Pb (II) to extracellular.	[151]
*Rhizobium metallidurans*	Cd (II)	Cd causes a conformational transition of EPS produced by *R. metallidurans* from larger spherical particles to smaller planar particles, thereby reducing damage to plant cells.	[118]
*Rhizobium tropici SRA1*	Zn/Hg/Mn/Mg/Co	FT-IR analysis of EPS indicated that the carboxyl group of uronic acid may be the binding site for divalent cations, contributing to the bridging between EPS and cations.	[152]
*Klebsiella sp.* J1	Pb (II)	Carboxylic acids, uronic acids, and esters in EPS combine with Pb (II) through ion exchange and complexation.	[153]
*Rhizobium tropici* CIAT899^T^	Al (III)	The higher the EPS yield, the higher the tolerance to Al (III).	[154]
*Rhizophagus irregularis* DAOM 197198	Cr (VI)	Under Cr (VI) stress, a large of EPS was generated on the surface of AMF to adsorb Cr (VI), Cr (VI) was reduced to Cr (III) in the cell wall, and Cr (III)-phosphate analogs were formed on the surface of AMF.	[145]
*Rhizophagus irregularis*	Cd (II)	After extra-root hyphae uptake and Cd transfer into root hyphae, Cd was mainly retained in the AMF structure and was not transmitted to plant cells.	[155]

**Table 3:** Effects of Rhizobium and AMF on plants in tripartite symbiosis in recent years.

Host Species	Type of Vaccination	Pollutants	Main Findings	Citations
*Medicago sativa*	*Sinorhizobium meliloti*, *Glomus mosseae*	Cd	Enhanced resistance of *M. sativa* to Cd stress after co-inoculation by increasing antioxidant enzyme activity and reducing plant photosynthetically-derived C partitioning into soil.	[186]
*Sesbania rostrata*	*Glomus etunicatum*, *Azorhizobium caulinodans*	Ur	Phytochelatase synthase (PCS) gene expression and organic acid content increased after co-inoculation of AMF with Rhizobium, which improved the phytoremediation efficiency of Ur.	[49]
*Anadenanthera peregrina*	*Acaulospora scrobiculata, Rhizobium BH-ICB-A8*	As	Co-inoculation of Rhizobium and AMF increased the growth and As-phytoremediation capacity of *A. peregrina*.	[29]
*Zea mays*	*Pseudomonas reactans EDP28*, *Rhizophagusregulations*	Cd/Zn	When co-inoculation increased root and stem biomass and stem elongation, Cd and Zn accumulation in maize tissues decreased.	[187]
*Glycine max*	*Glomus macrocarpum*, *Bradyrhizobium*	Pb	AMF enhances the uptake of Pb, which interferes with the establishment of a dual symbiotic relationship between AMF and Rhizobium and interferes with the survival of fungi in the soil.	[183]
*Sesbania cannabina*	*Glomus mosseae*, *Ensifer mexicanus*	PAHs	The triple symbiosis stimulates microbial development and soil enzyme activity to promote PAHs degradation.	[188]
*Medicago lupulina*	*Sinorhizobium meliloti, Agrobacterium tumefaciens*	Cu/Zn	Co-inoculation increased the growth and antioxidant activity of plants under Cu/Zn stress and thus enhanced the extraction capacity of metal plants.	[189]

## Data Availability

Not applicable.
